# Structural similarities of human and mammalian lipocalins, and their function in innate immunity and allergy

**DOI:** 10.1111/all.12797

**Published:** 2015-11-23

**Authors:** E. Jensen‐Jarolim, L.F. Pacios, R. Bianchini, G. Hofstetter, F. Roth‐Walter

**Affiliations:** ^1^ The interuniversity Messerli Research InstituteUniversity of Veterinary Medicine ViennaMedical University ViennaUniversity ViennaViennaAustria; ^2^ Institute of Pathophysiology and Allergy ResearchCenter of Pathophysiology, Infectiology and ImmunologyMedical University ViennaViennaAustria; ^3^ Biotechnology DepartmentCenter for Plant Biotechnology and GenomicsETSI MontesTechnical University of MadridMadridSpain; ^4^ Department of Natural Systems and ResourcesETSI MontesTechnical University of MadridMadridSpain

**Keywords:** allergen, animal, canine, iron, lipocalin

## Abstract

Owners and their domestic animals via skin shedding and secretions, mutually exchange microbiomes, potential pathogens and innate immune molecules. Among the latter especially lipocalins are multifaceted: they may have an immunomodulatory function and, furthermore, they represent one of the most important animal allergen families. The amino acid identities, as well as their structures by superposition modeling were compared among human lipocalins, hLCN1 and hLCN2, and most important animal lipocalin allergens, such as Can f 1, Can f 2 and Can f 4 from dog, Fel d 4 from cats, Bos d 5 from cow's milk, Equ c 1 from horses, and Mus m 1 from mice, all of them representing major allergens. The β‐barrel fold with a central molecular pocket is similar among human and animal lipocalins. Thereby, lipocalins are able to transport a variety of biological ligands in their highly conserved calyx‐like cavity, among them siderophores with the strongest known capability to complex iron (Fe^3+^). Levels of human lipocalins are elevated in nonallergic inflammation and cancer, associated with innate immunoregulatory functions that critically depend on ligand load. Accordingly, deficient loading of lipocalin allergens establishes their capacity to induce Th2 hypersensitivity. Our similarity analysis of human and mammalian lipocalins highlights their function in innate immunity and allergy.

## The current view on immune tolerance *vs* Th2 skewing of allergens

IgE‐mediated allergy is characterized by a Th2‐dominant immune response. The formed IgE may be directed against the genuine allergen or secondarily bind to molecules with similar epitopes on their surface, causing the phenomenon of cross‐reactivity. A limited number of allergen families can initiate Th2 responses strong enough to result in IgE formation in spite of sometimes having limited amino acid homologies. In these settings, functional similarity may be a dominant feature for their Th2 induction capacity.

Consequently, it is essential to understand the very first events that actually skew the immune response to Th2. Immune cells like NKT, eosinophils (that especially in the small intestine via CD103 DC activation control Th2 priming 1), basophils 2, nuocytes, and NKT cells 3, but also macrophages 4 are known sources of early IL‐4 and IL‐13. These cytokines are released quickly upon stimulating the cells under involvement of a Notch‐regulated CNS‐2 enhancer 5 and the events are connected to memory in CD4 T cells 2. Disturbances of the skin and mucosal barriers and subsequent TSLP release may be crucial upstream events 6. One explanation could be that some allergens have enzymatic functions and thereby produce an epithelial danger signal 7, but many allergens have no proteolytic activity and do still exert ‘adjuvant‐like activation’ 8. It is thus not entirely clear which danger signals go along with allergens that initiate priming or triggering of these cells to release Th2 cytokines, and would thereby mark allergens as *special*. It is noteworthy that among the known protein families in the 2009 Pfam database only 1.4% are considered allergens 9. In the case of respiratory mammalian allergens a few protein families are absolutely dominant: lipocalins, secretoglobins and kallikreins 10, and IgE levels against them correlate with the severity of asthma 11.

## Animal keeping: who is at risk?

The question of whether interactions with animals provide protection or increase risk is hotly debated, with particular points of discussion being the subject's age at time of exposure, and the exact animal species or breed. In German‐speaking Europe between 16% and 25% of households own cats, and 12% dogs (Table 1), with an estimated dark number of plus 30%. The interrelation between people and domestic animals has thus considerably changed in the last decades. Humans and pets in Western societies are cohabitating more close and in a cleaner way than ever before, a development which is associated with a good economy. Simultaneously, the rates of allergies and asthma in human patients caused by animal dander are increasing 12. In a cohort study of 696 school children 259 were sensitized to animals, and the majority of them to multiple species 13. The higher awareness today of allergy risk associated with pet ownership and the desire for nonfurry pet animals go hand in hand, introducing novel, alternative allergen threats into homes such as those presented from feed insects for reptiles 14. However, pets may also shuttle traditional allergens into homes: house dust mites can be identified in bedding, skin and hair coats of dogs 15. Interestingly, also pet animals and horses increasingly suffer from atopic eczema and other forms of allergies 16, 17. Therefore, it might be important to look at the problem bi‐directionally and watch for systematic problems in our society and environment.

**Table 1:** Statistical comparison of percentage and numbers of animals in German‐speaking households (Statistics Germany http://www.wissenswertes.at/index.php?id=haustiere-statistik)

Species	Austria (%)	Switzerland (%)	Germany (%)	Numbers in Germany (Mio.)
Cats	25.6	25	16.5	8.2
Dogs	30	12	13.3	5.4
Rodents		11	6.4	5.6
Birds		7	4.9	3.4
Aquaria		6	4.4	2.0
Ponds			4.0	2.1
Terraria			1.2	0.4

In contrast to the earlier trend to minimize allergen release by washing pets 18, most recent work suggests that the introduction of pets into homes early in life is protective 19. It has further been shown that lowering pet exposure may reduce the IgE and symptom levels if allergy is established 20. The interplay of allergens and the microbiome may be decisive for inducing hypersensitivity: Low levels of Firmicutes and Bacteriodetes in the skin flora concomitant with high allergen exposure enhance the risk for atopy and asthma 21. Needless to say that forced hygiene, washing and disturbance of the skin pH, enzymatic shampooing 7, and even the water quality 22, may impact the physiologic skin flora 23. In some studies cat and dog ownership did not elevate the risk of asthma 24, which might be correlated with exposure to the associated ‘good’ microbiomes of the animals. However, people with dog allergy more seldom keep dogs 25.

The observation that there are breed‐specific differences in eliciting symptoms has initiated the search for ‘hypoallergenic’ cats, dogs, and horses and stimulates breeders to single out mutants. The experimental evidence, however, is sparse and this topic needs to be addressed in its whole complexity regarding the countless breeds of domestic animals. The situation gets even more complicated as an animal's age and gender also play a role in allergies to them 26. Here, molecular diagnosis has significantly contributed to important practical recommendations 19, including the neutering, or avoidance of male animals 27, 28. Whereas the expectations were that hypoallergenic animals would shed less allergens, the contrary turned out to be the case: in a recent study putative hypoallergenic dogs produced higher levels of Can f 1, a lipocalin allergen 29.

## Comparing animal, human, and other lipocalins

What is not yet understood is why allergens in particular provoke a Th2 shift in the immune response and lead to IgE production. Why exactly, are lipocalins so important elicitors of allergies to animals even though humans also do express lipocalins, a fact that is maybe less known to the allergist?

**Figure 1:**
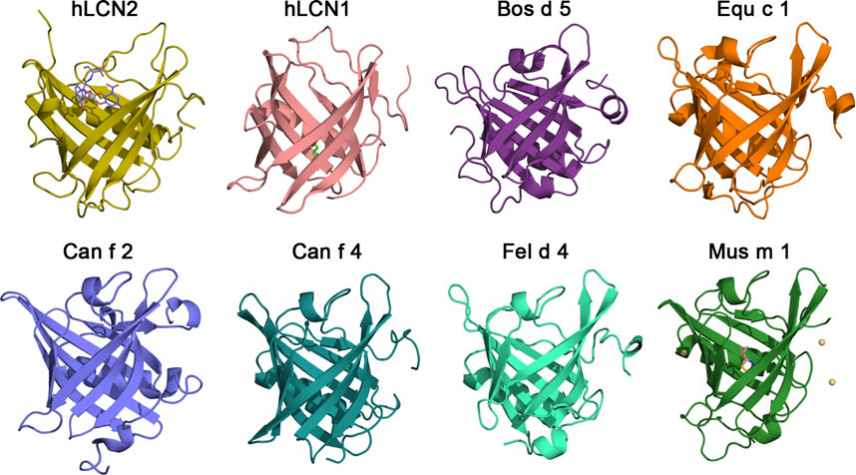
Gallery of human and animal lipocalins. PDB codes for experimental structures are given in parentheses. hLCN2: human LCN2 in complex with the siderophore enterobactin shown as sticks chelating the central Fe^3+^ ion shown as a gray sphere (1L6M 82). hLCN1: human tear lipocalin in complex with 1,4‐butanediol (3EYC 83). Bos d 5: bovine β‐lactoglobulin unliganded form (3NPO 84). Equ c 1: major horse allergen (1EW3 85). Can f 2: dog allergen (3L4R 45) with IgE cross‐reactivity with the cat allergen Fel d 4. Can f 4: dog dander allergen (4ODD 86). Fel d 4: cat allergen, homology model structure based on template Equ c 1. Mus m 1: pheromone binding rodent major urinary protein in complex with 2‐(sec‐butyl)‐thiazole and Cd^2+^ ions shown as yellow spheres (1MUP 87).

Lipocalins owe their name to the fact that they usually carry lipids (or other hydrophobic compounds) accommodated in a large, calyx‐like cavity formed by their characteristic β‐barrel fold (Fig. 1). Ligand specificity of lipocalins is regulated by the amino acid residues that line their binding pocket. A large variety of ligands, most of them hydrophobic or amphiphilic, binds to lipocalins that include lipids, steroids, hormones as well as metabolites such as vitamins, cofactors, and odorants. In some cases, binding of the ligand is critical for the biological functions of the lipocalin.

Exploring the PDB by entering the query ‘lipocalin’, renders nearly 300 experimental structures available for lipocalins belonging to 24 eukaryotic species and 10 bacteria species of which 79 structures correspond to humans (Table 2).

**Table 2:** Organisms with experimental structure of lipocalins available in the Protein Data Bank

Organism		Organism	
Eukaryota	Number of entries	Bacteria	Number of entries
*Anguilla japonica*	3	*Bacillus pumilus*	1
*Arabidopsis thaliana*	2	*Bacillus subtilis*	1
*Argas monolakensis*	4	*Bacteroides eggerthii*	1
*Argas reflexus*	2	*Bacteroides ovatus*	1
*Bos taurus*	37	*Bacteroides thetaiotaomicron*	2
*Canis lupus familiaris*	2	*Bacteroides uniformis*	3
*Capra hircus*	3	*Bradyrhizobium diazoefficiens*	3
*Coturnix coturnix*	1	*Enterococcus faecalis*	1
*Coturnix japonica*	2	*Escherichia coli*	6
*Diploptera punctata*	3	*Escherichia coli K‐12*	2
*Equus caballus*	1	*Helicobacter pylori*	1
*Homarus gammarus*	4	Total	22
*Homo sapiens*	79		
*Lingulodinium polyedrum*	1		
*Mesocricetus auratus*	1		
*Mus musculus*	34		
*Ornithodoros moubata*	4		
*Ovis aries*	2		
*Pieris brassicae*	5		
*Rattus norvegicus*	4		
*Rhipicephalus appendic*	6		
*Rhodnius prolixus*	68		
*Sus scrofa*	6		
*Trichosurus vulpécula*	3		
Total	277		

As of June 22, 2015, the search for ‘*lipocalin*’ in the PDB yielded 299 entries distributed between eukaryota and bacteria.

However, these numbers include mutants, engineered variants and the same protein in complex with different ligands, particularly for the most studied cases such as bovine, murine, and human lipocalins.

Allergenic lipocalins have been especially identified in furry animals 19, for instance in dogs: Can f 1, ‐2, ‐4, ‐6, cats: Fel d 4, ‐7, mice: Mus m 1, horses: Equ c 1, guinea pigs: Cav p 2, ‐3 30, and hamsters 31. Lipocalins are also found in tick saliva 32 where they sequester histamine and thereby interfere with its signaling via H1 receptor 32, and contribute to camouflaging the bite. Importantly, humans also express ‘a menagerie of lipocalins’ 33, such as LCN2 (alternatively called siderocalin/SCN; neutrophil gelatinase‐associated lipocalin/NGAL, 24p3), or human LCN1 (alias tear lipocalin, TLC). A gallery of human and animal lipocalins for which experimental structures exist is represented in Fig. 1. Their well‐recognized characteristic β‐barrel fold has emerged in the last few years as a successful molecular scaffold to engineer novel binding proteins, so called anticalins, with human lipocalins playing a particularly relevant role in clinical applications 33. In an innovative approach four different canine lipocalins were recently linked into one multimeric molecule which indeed induced IgG upon vaccinating mice and which therefore may be used for vaccination against dog allergy in the future 34.

Lipocalins occur in secretions such as saliva, in urine (besides kallikreins) 28, and shed skin, which are all important sources for the release of allergens into our environment 35. Taken the intense interactions between owner and animal it is plausible that there is a bidirectional exchange of allergens, as it is the case with phylotypes of the microbiome of human dog owners and dogs 36.

Given their importance, many lipocalins of domestic animals and pets have been cloned and their production improved (such as Can f 1, ‐2, or ‐4 37, 38, 39, 40), but methods were also developed for the quantification of the natural allergens to be able to evaluate the exposure levels in different environments, e.g., for Can f 4 41 which is important for over 80% of people with dog allergies for IgE binding. Such measurements have recently brought up the fact that so‐called hypoallergenic dogs shed even higher levels of Can f 1 into homes 29.

## Comparing structural features of lipocalins

Lipocalins display a well‐known overall similarity in their fold in spite of the relatively low degree of amino acid sequence identities of below 25% 42. This fold is characterized by a calyx‐ shaped cavity within the central β‐barrel formed by eight antiparallel β‐strands βA‐βH (in what follows, labels refer to Fig. S1). Loops around the cavity regulate ligand access to the calyx binding site. The extended loop L1 which comprises a 3_10_(2) helix together with hairpin loops L3, L5, and L7 define the top wider end of the barrel, whereas hairpin loops L2, L4, and L6 make the bottom narrower end. This arrangement settles a topology of the β‐barrel which is one of the distinctive features of lipocalins. This fold also presents a conserved N‐terminal 3_10_(1) helix as well as a medium‐size (about 10 residues) conserved α helix H1 and an additional β‐strand, βI, near the C‐terminal segment. Some lipocalins (e.g., bovine lactoglobulin) display additional secondary structure elements such a small N‐terminal β‐strand (β) and a C‐terminal 3_10_(3) helix. The presence of one to three structurally conserved regions (SCR1–SCR3) associated to particular local arrangements of strands and helices have been used to divide lipocalins into two structural subfamilies termed kernel lipocalins (those having the three SCRs) and outlier lipocalins (those having a maximum of two SCRs). SCR1 comprises 3_10_(1) and βA in the N‐terminal region, SCR2 is composed of sections of βF and βG together with loop L6, and SCR3 is defined by the end of βH together with adjacent residues of H1 in the C‐terminal region (Fig. S1). Obviously, amino acid residues in loops L1, L3, L5, and L7 lining the binding site regulate the ligand specificity of lipocalins. Although the name of this protein family makes explicit mention of lipophilic (hydrophobic) compounds, there is a large variety of ligands including amphiphilic molecules or compounds with hydrophobic segments also bearing polar groups that bind to lipocalins.

All these structural similarities observed in these proteins could help to understand IgE cross‐reactivities 43 between animal and human lipocalins, and between lipocalins from different animals 44, 45, 46, although not necessarily correlating with the symptoms.

**Figure 2:**
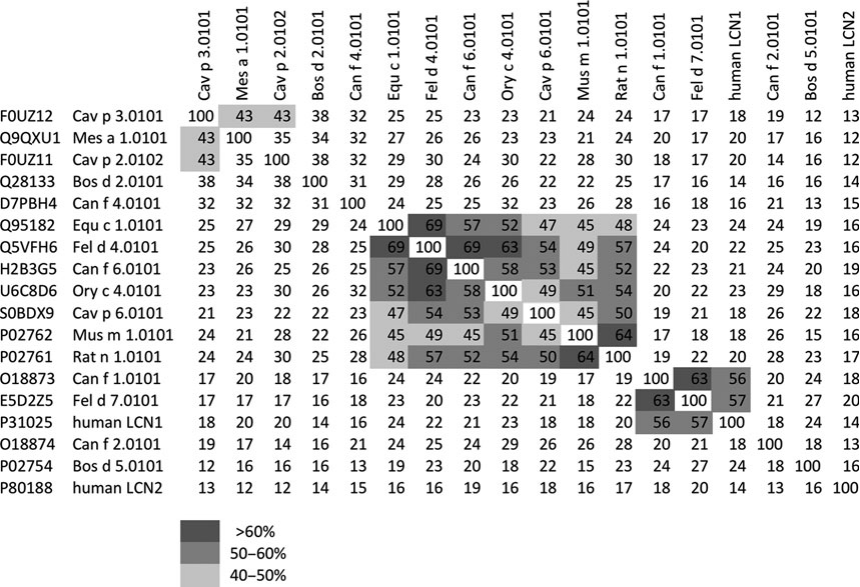
Comparison of human lipocalins and major animal lipocalin allergens. Amino acid identities (%) between mammalian lipocalin allergens and human lipocalin 1 and 2 were determined with Clustal Omega via http://www.ebi.ac.uk/Tools/msa/clustalo/. Highlighted regions show identities higher than 40%. The different gray shades illustrate the levels of amino acid identities. Amino acid sequences were retrieved from UniProtKB database (http://www.uniprot.org/uniprot/).

For instance, in the current view at the amino acid level the dog allergens Can f 1 and Can f 2 correspond to human LCN1 and ‐2, respectively, whereas the four molecules only share 25% amino acid similarities between them. The amino acid sequences of various animal and human lipocalins LCN1 and ‐2 are aligned in Fig. 2. Identities above 60% occur between Fel d 4 and Equ c 1, Fel d 4 and Can f 6, Ory c 4, Rat n 1 and Mus m 1, and Can f 1 and Fel d 7.

In contrast, there was no amino acid homology among human LCN2 and any of the analyzed animal lipocalins, only 57% among human LCN1 and Fel d 7, and 56% among LCN1 and Can f 1.

T‐cell responses have been found to be ‘suboptimal’ for Can f 1 47, although resembling those to human LCN1 48. Can f 4 T‐cell responses were shown to be a Th2‐deviated memory response, in part to peptides with promiscuous HLA‐binding capacity 49, whereas the DR4–DQ8 haplotype and T‐cell receptor Vbeta T‐cell subset were indicative for the absence of Can f 1 allergy 50.

**Figure 3:**
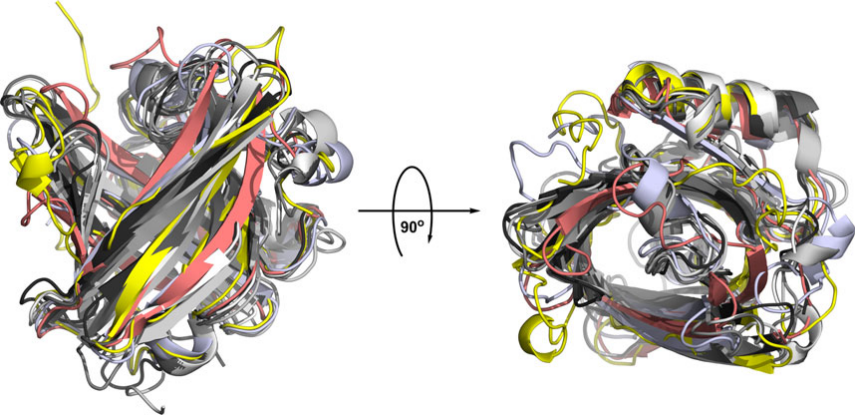
Symphony of human and animal lipocalins by structural superimposition. Human LCN2 and LCN1 lipocalins are colored yellow and salmon, respectively, Bos d 5 is colored blue white and the remaining lipocalins in Fig. 1 are colored in different shades of gray from white (Equ c 1) to black (Mus m 1). The rotated image on the right gives an upper view of the calyx‐like cavity.

The above implies that amino acid sequence identities alone do not sufficiently explain why all lipocalins are important in innate immune regulation, and why they are typically allergenic. The known anti‐bacterial activity of the human neutrophil lipocalin LCN2 (NGAL) is caused by the high affinity of this lipocalin for the siderophore‐Fe3+ complex and it should be investigated systematically whether from this it can be extrapolated to animal lipocalins. In fact, the molecular ligand transport function of lipocalins and of lipocalin‐like proteins could be particularly significant for the Th2 skewing capacity, independent of their mammalian 51 or plant 52 origin. We propose that this feature is likely connected with the similar β‐barrel fold in human and animal lipocalins (Fig. 3) 33, as well as with the binding plasticity of their calyx‐like cavity able to accommodate a great disparity of substances involved in metabolism and communication 33, 39, 53. Major sources of (at least the human) LCN2 are the liver 54 and macrophages 55, with sphingosin‐1‐phosphate inducing its production. Lipocalins are elevated and released during inflammation 56, infections 57 and sepsis 58, 59, 60, especially when the urinary tract is affected in humans and dogs 61, 62; in cardiovascular inflammation they get overexpressed in the brain and linked with depression 63; in cancer LCN2 is overexpressed 11, 64 prompting interaction with metalloproteinase‐9 (MMP‐9) 65, but only LCN2 is prognostic 66. In the immunosuppressive environment of the tumor stroma LCN2 cooperates with CCL2 to induce immunoregulatory DCs and subsequently CD4(+)FoxP3(+) Treg cells and supports metastasis 67. Moreover, local IL‐10 stimulates tumor infiltrating M2 macrophages to release LCN2 and iron 68, 69. Hence, in cancer the opposite of what is needed in allergy is the problem 70. It is further known that macrophages convert to an anti‐inflammatory type by IL10 and glucocorticoid stimulation 71. Alternatively activated macrophages further regulate the immune response by secreting LCN2, which is directly linked with IL10 formation (and in our own hands glucocorticoid release). Taken together, lipocalins are regarded as biomarkers for several diseases 72 and are at the crossing point between immunity and tolerance that is of particular interest for allergen immunotherapy.

## The secret function of lipocalins determined by their pocket load

Some important human lipocalins are considered acute phase proteins with innate immune function, especially against gram‐negative bacteria. Their growth depends on iron, which they secure via the secretion of a bacterial siderophore, [= a high‐affinity iron acquisition molecule], like enterobactin, an iron‐specific chelating agent closely related to the ligand in the crystal structure of human LCN2 displayed in Fig. 1. We speculate that, like LCN2 which withdraws iron from bacteria by accepting it into their own pocket, also some of the other mammalian lipocalins may act bacteriostatically (Fig. 3) 73. On the other side of this evolutionary interplay, bacterial siderophores can inhibit the function of neutrophil myeloperoxidase and enhance the bacterial survival 74, or simply overwhelm the lipocalin defense system 75. A lipocalin‐2 receptor (termed 24p3/NGAL) and Lipoprotein Receptor‐Related Protein have been described binding LCN in humans and rodents 65, 76, possibly also involving MMP9 in a triple interaction.

**Figure 4:**
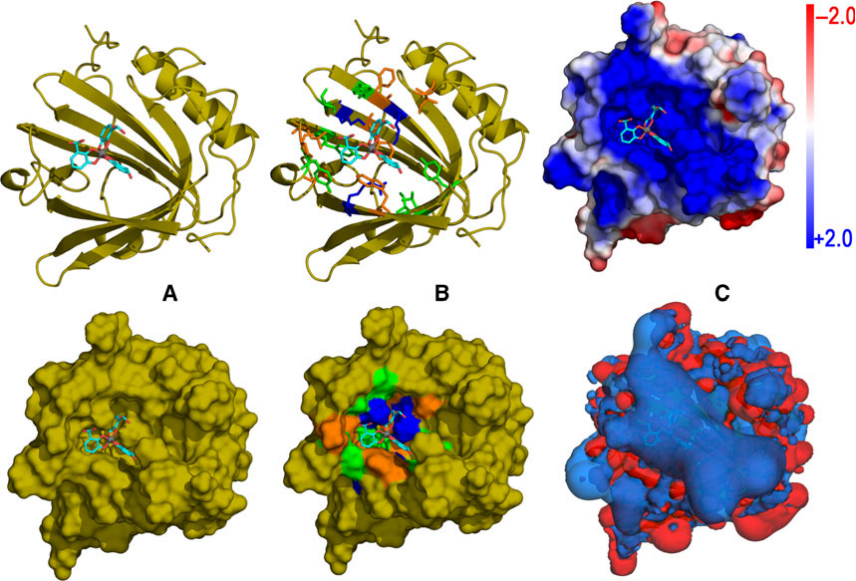
Prototype of lipocalin‐siderophore binding: model complex of human LCN2 and Fe(DHBA)_3_, a hexadentate iron‐chelator. The siderophore (DHBA = 2,3‐dihydroxybenzoic acid) is depicted as sticks with carbons in cyan, oxygens in red, and Fe^3+^ ion as a gray sphere. (A) Geometry of the siderophore in the cup‐like cavity (top) and the corresponding protein surface showing the topography of the pocket (bottom). (B) Residues within a 5 Å distance from the ligand color‐coded as follows: basic = blue, polar = green, and nonpolar = orange. Top: ribbon and sticks diagram. Bottom: the corresponding color‐coded surface. (C) Poisson–Boltzmann (PB) electrostatic potential of hLCN2. Top: PB potential mapped onto the protein surface according to the scale indicated in the bar. The strongly positive electrostatic nature of the pocket area stabilizes the electrically negative oxygenated groups in the siderophore. Bottom: +2 (blue) and ‐2 (red) 3D isosurfaces of the PB electrostatic field created by hLCN2. A large positive isosurface covers the cavity area in which the siderophore is accommodated.

Less attention has been given to a potentially differential immune function of lipocalins depending on the load state of their pocket, either holo – full, or apo – empty. Interestingly, the major birch pollen allergen Bet v 1 has a relation to bacterial proteins 77 by its old scaffold and a central pocket, which was predicted to bind quercetin‐3‐*O*‐sophoroside 78. However, the flavonoid quercetin also behaves as a siderophore 52. By means of an *in silico* approach using flexible docking calculations and geometry optimizations we were able to analyze the optimal binding of human LCN2 to several siderophore‐iron complexes with 1, 2, or 3 catechol chelating moieties 79. This study allowed us to estimate protein‐ligand dissociation constants in the micro‐ to nanomolar range indicative of a high affinity that arises to a great extent from the electrostatic interactions between amino acid residues surrounding the outer part of the cup‐like cavity and polar groups of the siderophore 79. A further illustration of this optimal binding is depicted in Fig. 4 where the model complex between hLCN2 and a hexadentate iron chelator, Fe(DHBA)_3_, is shown. Note that the presence of hydrophobic moieties and polar groups in the ligand is consistent with the presence of hydrophobic residues together with charged (mainly basic) side chains in the protein that produce the electrostatic effects largely responsible for this efficient protein‐ligand binding. However, we emphasize that more experimental studies have to be undertaken not only to approve our estimation with certainty *in vitro*, but also to be able to extrapolate to other lipocalin allergens.

More importantly, the load of this binding pocket is decisive for the immunomodulatory function of lipocalins. Only the apo‐form of lipocalin Bos d 5 and lipocalin‐like allergen Bet v 1 51, 52 promoted the survival of CD4+ T cells and IL‐13 production in human PBMCs, whereas the holo‐form was nonallergenic, so far at least *in vitro*. Consistent with this function, the loading state of brain lipocalin is decisive for the survival of neuronal cells 80. Furthermore, in a dose‐dependent loading approach, holo‐LCN2 had the highest capacity to induce HLA‐G(+)/FoxP3(+) T‐regulatory cells 81. Therefore, it may be anticipated that exogenous lipocalins, animal allergens, may be tolerated rather than inducing allergy, when complexed with ligands.

## Synopsis

Humans and their domestic animals share molecules that play a role both physiologically in innate immunity, and pathologically as allergens. Whereas many important animal allergens relevant for humans belong to the lipocalin family, it is not known yet whether *vice versa* human lipocalins may act as allergens for pet animals. It is not by accident that the lipocalin fold is highly conserved in the animal kingdom. It allows lipocalins to withdraw iron from microbes into their molecular pocket and thereby act bacteriostatic.

In conclusion lipocalins may, independent of their mammalian other animal, or plant origin, house siderophore ligands, which critically determine their innate immunomodulatory as well as allergenic characteristics. It has been shown that in loaded state human lipocalins induce regulatory T cells, whereas empty lipocalins rather promote Th2 responses and inflammation. To this end, the interplay between exogenous lipocalins and human LCNs is not resolved, but it is tempting to speculate that they interfere with each other either via receptor binding or via capturing each other's iron ligand.

## Author contributions

Jensen‐Jarolim E responsible for scientific concept, writing of the manuscript, and literature search. Pacios Luis F performed calculations, computer modeling, and structural alignments of all structures of this study (Figs 1, 3 and 4). Bianchini R and Hofstetter G involved in amino acid sequence searches and comparisons and contributed to the writing of the manuscript; Hofstetter G also contributed to the design of Fig. 2. Pacios, LF, and Roth‐Walter, F contributed to the overall scientific concept and writing of the manuscript.

## Conflicts of interest

Erika Jensen‐Jarolim, Luis F. Pacios and Franziska Roth‐Walter declare that they are inventors on PCT/EP2015/050126. Rodolfo Bianchini and Gerlinde Hofstetter declare no conflict of interest.

## Supporting information


**Figure S1.** 3D structure and topology of a prototypical lipocalin.
