# Supplementary material for: Structural similarities of human and mammalian lipocalins, and their function in innate immunity and allergy
Source: Allergy. 2015 Nov 23;71(3):286–94. doi: 10.1111/all.12797 (PMC4949658; doi:10.1111/all.12797)
Supplement: Supplementary file 1 — Figure S1. 3D structure and topology of a prototypical lipocalin. [file ALL-71-286-s001.docx]

**Supplementary Figure 1:**

**
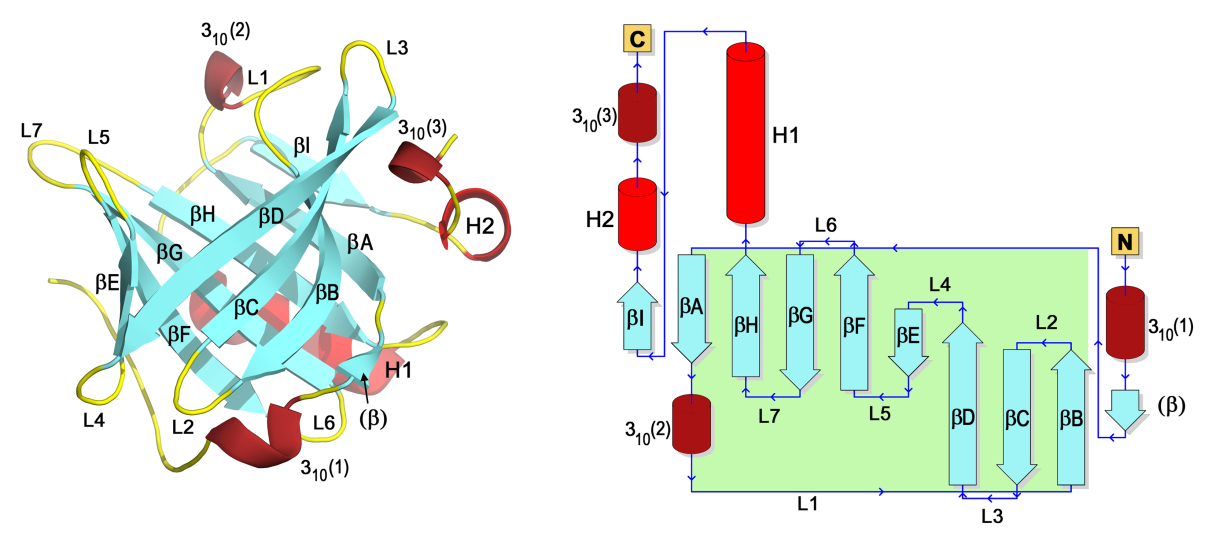
**

**Legend to Supplementary Figure 1:**

**3D structure and topology of a prototypical lipocalin.** *Left*: ribbon diagram of the 3D structure of bovine β-lactoglobulin unliganded form (Bos d 5 allergen, PDB code 3NPO). β-strands are colored in cyan, α-helices in light red, 3_10_ helices in deep red, and loops in yellow. Secondary structure assigned with DSSP (1, 2). *Right*: topological diagram of this structure edited from the diagram obtained with PDBSum (3) keeping the same labeling and coloring used in the 3D image on the left. The central β-barrel domain defined by the eight strands βA-βH is marked with a green rectangle.

Data S1 References

1. Kabsch W, Sander C. Dictionary of protein secondary structure: pattern recognition of hydrogen-bonded and geometrical features. *Biopolymers* 1983;**22**(12):2577-2637.

2 Touw WG, Baakman C, Black J, te Beek TA, Krieger E, Joosten RP, et al. A series of PDB-related databanks for everyday needs. *Nucleic Acids Res* 2015;**43**(Database issue):D364-368.

3 de Beer TA, Berka K, Thornton JM, Laskowski RA. PDBsum additions. *Nucleic Acids Res* 2014;**42**(Database issue):D292-296.
